# Red-light excited efficient metal-free near-infrared room-temperature phosphorescent films

**DOI:** 10.1093/nsr/nwab085

**Published:** 2021-05-11

**Authors:** Siyu Sun, Liangwei Ma, Jie Wang, Xiang Ma, He Tian

**Affiliations:** Key Laboratory for Advanced Materials and Feringa Nobel Prize Scientist Joint Research Center, Frontiers Science Center for Materiobiology and Dynamic Chemistry, School of Chemistry and Molecular Engineering, East China University of Science and Technology, Shanghai 200237, China; Key Laboratory for Advanced Materials and Feringa Nobel Prize Scientist Joint Research Center, Frontiers Science Center for Materiobiology and Dynamic Chemistry, School of Chemistry and Molecular Engineering, East China University of Science and Technology, Shanghai 200237, China; Key Laboratory for Advanced Materials and Feringa Nobel Prize Scientist Joint Research Center, Frontiers Science Center for Materiobiology and Dynamic Chemistry, School of Chemistry and Molecular Engineering, East China University of Science and Technology, Shanghai 200237, China; Key Laboratory for Advanced Materials and Feringa Nobel Prize Scientist Joint Research Center, Frontiers Science Center for Materiobiology and Dynamic Chemistry, School of Chemistry and Molecular Engineering, East China University of Science and Technology, Shanghai 200237, China; Key Laboratory for Advanced Materials and Feringa Nobel Prize Scientist Joint Research Center, Frontiers Science Center for Materiobiology and Dynamic Chemistry, School of Chemistry and Molecular Engineering, East China University of Science and Technology, Shanghai 200237, China

**Keywords:** amorphous metal-free phosphorescence, near-infrared phosphorescence, visible light excitation, phenolsulfonphthaleine derivatives, half-subtractor

## Abstract

A set of red-light-excited, metal-free room-temperature phosphorescence (RTP) systems was constructed with brominated phenolsulfonephthaleine derivatives. The best metal-free RTP system has the reddest near-infrared (NIR) RTP emission (λ_p_ = 819 nm) with the highest phosphorescence quantum yield (Φ_RTP_ = 3.0%) so far identified. The RTP emission can be switched ON-OFF by adding acid and alkali alternately. A logic operation with half-subtractor function and dual-channel response (visible light emission/NIR RTP emission) was also constructed based on these properties.

## Teaser

Stimulus-responsive organic near-infrared room temperature phosphorescent film materials were constructed, and a corresponding phosphorescent half-subtractor logic operation conducted as well.

## INTRODUCTION

Having unique luminescence characteristics [1–5], metal-free room-temperature phosphorescence (RTP) has gained widespread attention in mechanisms [6–8] with potential applications in the fields of optoelectronic devices [9,10], biological imaging [11] and the like [12,13]. Traditionally, in design of metal-free RTP molecules, heavy atom effects and n→π* transition are integral in promoting the intersystem crossing (ISC) process from the molecular level. Essentially, the environment in which molecules are located plays a decisive role in generating RTP. This is because a rigid external environment may effectively inhibit the non-radiative relaxation at its triplet excited state [14], and the environment's oxygen barrier efficiency/ability may also inhibit the triplet-triplet energy transfer process between the phosphors and the triplet oxygen to reduce the non-radiative relaxation of such triplet excitation state [15,16]. A review of the literature provides recent reports on construction of metal-free RTP systems in the crystalline state [17–20], solution state [21,22] and amorphous state [23,24], among others [7,10,25–28]. The visible-light-excited RTP system can effectively avoid photobleaching and realize excellent applications in biological imaging, although use of a visible-light-excited RTP emission system is relatively rare [21,29–31]. It is difficult to obtain the spontaneous radiation of organic materials in the near-infrared (NIR) region because of the energy gap law for radiationless transition [32–34]. In 2019, BODIPY derivatives were used to construct a visible-light-excited infrared RTP system [29], although difficulty and inefficiency were reported in preparing RTP materials in the near-infrared region. In the present study, a set of metal-free RTP materials with the highest NIR RTP quantum yield (Φ_RTP_ = 3.0%) and the reddest emission (λ_p_ = 819 nm) were prepared from commercially available raw materials (PVA and brominated phenolsulfonephthaleine dyes), based on which a logic operation with half-subtractor function and dual-channel response (visible light emission/NIR RTP emission) was constructed.

## RESULTS AND DISCUSSION

**Figure 1.**
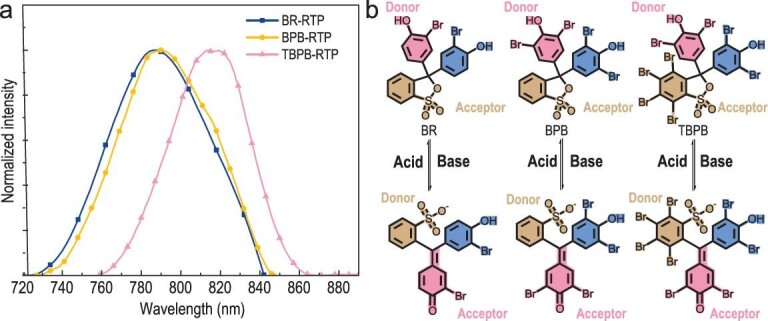
(a) RTP emission of PSP@PVA-N (λ_ex_ = 550 nm). (b) Structural transformation of BR, BPB and TBPB in aqueous solution with differing pH values and their charge distribution in the ground state (S_0_). The specific color regions (orange, dark pink and blue) correspond to specific functional groups of molecules.

Doping chromophores in polyvinyl alcohol (PVA) rigid matrix to induce RTP has proved to be an effective strategy to construct amorphous RTP materials. PVA matrix can effectively inhibit the non-radiative relaxation of the chromophore and insulate oxygen to protect the triplet excited state of the chromophore. In the present study, phenolsulfonephthaleine (PSP) derivatives were doped into a PVA matrix to construct a series of amorphous metal-free films with near-infrared RTP emission (Fig. 1a). Three PSP derivatives (Fig. 1b) were selected to construct amorphous materials with RTP emission in the PVA matrix at a concentration of 1 wt% (BR@PVA, BPB@PVA and TBPB@PVA). XRD spectra proved that all films in differing conditions were amorphous (Figs S1 and S2). The preparation methods for these materials are detailed in the Method section.

Previous studies revealed that these PSP derivatives are highly sensitive pH indicators in water [35] and have different molecular structures in acidic and neutral aqueous solutions. In particular, PSP is ring-closed under acidic conditions, while under neutral and alkaline conditions, it will lose protons and open the ring [35].

**Figure 2.**
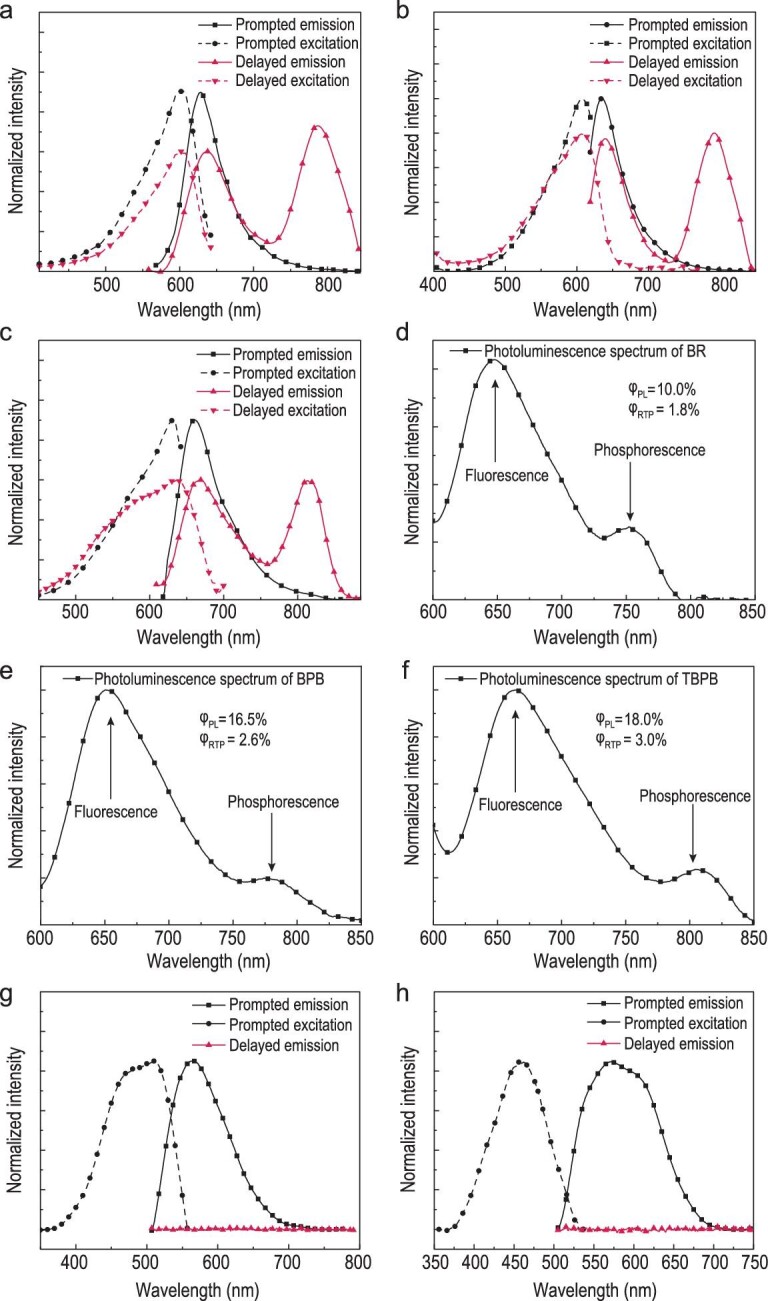
Photophysical properties of near-infrared RTP materials. Prompted fluorescence spectra, delayed emission spectra and the corresponding excitation spectra of (a) BR@PVA-N (λ_ex_ = 550 nm, λ_em, prompted_ = 650 nm, λ_em, delayed_ = 800 nm), (b) BPB@PVA-N (λ_ex_ = 600 nm, λ_em, prompted_ = 650 nm, λ_em, delayed_ = 800 nm) and (c) TBPB@PVA-N (λ_ex_ = 600 nm, λ_em, prompted_ = 650 nm, λ_em, delayed_ = 800 nm). Steady state photoluminescence spectra of (d) BR@PVA-N (λ_ex_ = 550 nm), (e) BPB@PVA-N (λ_ex_ = 550 nm) and (f) TBPB@PVA-N (λ_ex_ = 550 nm). Prompted fluorescence spectra, delayed emission spectra and the corresponding excitation spectra of (g) BR@PVA-H (λ_ex_ = 450 nm, λ_em_ = 600 nm) and (h) BPB@PVA-H (λ_ex_ = 450 nm, λ_em_ = 600 nm).

Figure 2 shows the emission and excitation spectra of these PSP-doped PVA films in various states, with detailed photophysical information presented in Table 1. It can be observed that PSP derivatives in differing states are manifested with different structures, and RTP emission in PVA is shown in Fig. 2. The open-ring and closed-ring states of PSP derivatives in the PVA matrix were first evaluated by the absorption spectra of the obtained PSP@PVA films at different pH values. Noticeably, the UV-Vis absorbance of PSP@PVA prepared under acidic conditions (PSP@PVA-H) at 600 nm was significantly lower than that prepared under neutral conditions (PSP@PVA-N; Figs S3–S5). Although the color doped in PVA remained the same as that in the aqueous solution, it changed according to acidity [35]. The dried PSP@PVA film and PSP aqueous solution looked colorless or light-yellow in an acidic environment, whereas the color of the dried PSP@PVA film and PSP aqueous solution turned dark blue in a neutral or alkaline environment (Fig. S6). Therefore, it can be inferred that the PSP molecules, which were doped in PVA-H films, were in a closed-ring state, while the molecules in PVA-N were in an open-ring state. Additionally, the open-ring (PSP@PVA-N)/closed-ring state (PSP@PVA-H) of PSP derivatives can be controlled *in situ* by adding acids and bases to the PVA matrix.

**Table 1.** Photophysical properties of BR@PVA, BPB@PVA and TBPB@PVA.

Compound	λ_ex_ [nm]	λ_em_ (f) [nm]	λ_em_ (TADF) [nm]	λ_em_ (P) [nm]	τ_P_ [ms]	Φ_PL_ [%]	Φ_RTP_ [%]
BR@PVA-N	603	630	635	789	0.72	10.0	1.8
BPB@PVA-N	606	632	637	793	0.58	16.5	2.6
TBPB@PVA-N	634	660	665	819	0.27	18.0	3.0
Reference	540	565	–	770	0.71	–	0.5

F: fluorescence; TADF: thermally activated delayed fluorescence; P: RTP. The reference is the photophysical properties of reported metal-free NIR RTP materials.

As can be seen in Fig. 2a–f, the delayed fluorescence emission and moderate RTP emission were obtained by these three films in neutral conditions. Channel splitting emission spectra of the prompted and delayed emission of PSP@PVA show that they shared the same Franck–Condon excited state as the origin of the emission, which was further demonstrated by the effective overlap of their corresponding excitation and UV-Vis absorption spectra (Figs S3–S5). Firstly, the RTP properties of PSP@PVA films were studied. The peaks of the prompted fluorescence emission of these films were primarily red emission, located mainly between 600 nm and 700 nm (Fig. 2a–c). The delayed emission, instead, peaked around 600–700 nm, overlapping completely with that of the prompted fluorescence emission. Furthermore, as shown in Fig. 2a–c, a new emission peak with a large Stokes shift was observed above 750 nm. Among these observable emissions, the peaks of the delayed emission located in the near-infrared region with large Stokes shifts were classified as RTP emission peaks. These PSP@PVA-N films exhibited near-infrared RTP emission with moderate phosphorescence quantum yields (Φ_RTP _= 1.8–3.0%), which can be further demonstrated by their steady-state photoluminescence spectra (Fig. 2d–f). The delayed emission located at 600–700 nm, which is on a par with the fluorescence emission location and shape of the prompted emission, was classified as thermally activated delayed fluorescence (TADF) emission. According to the spectral data, the lifetime of the TADF was shorter than RTP emission, which is consistent with the differences between TADF and RTP (Figs S7–S9, Table S1). The frontier orbital analysis of the open-ring state further verified the rationality of the observation of TADF from a bromide-substituted PSP derivative in a rigid matrix environment [36].

However, only the prompted fluorescence emission could be observed in the closed-ring PSP derivatives in the PVA matrix, and no observable signals were shown on the delayed emission spectra. It was observed that the absorption spectra of open-ring and closed-ring PSP derivatives in the PVA matrix had significant changes (absorption peak and changes in shapes). These changes were attributed to the decrease in conjugation of PSP derivatives, which converted from a ring-open state to a ring-closed state. The prompted fluorescence of closed state BR@PVA-H and BPB@PVA-H films had a significant blueshift (∼150 nm), compared with the prompted emission in open-ring states. Besides, the emission of the TBPB@PVA-H film was quenched (Fig. S10), and its absorbance with a wavelength greater than 300 nm was also very weak.

**Figure 3.**
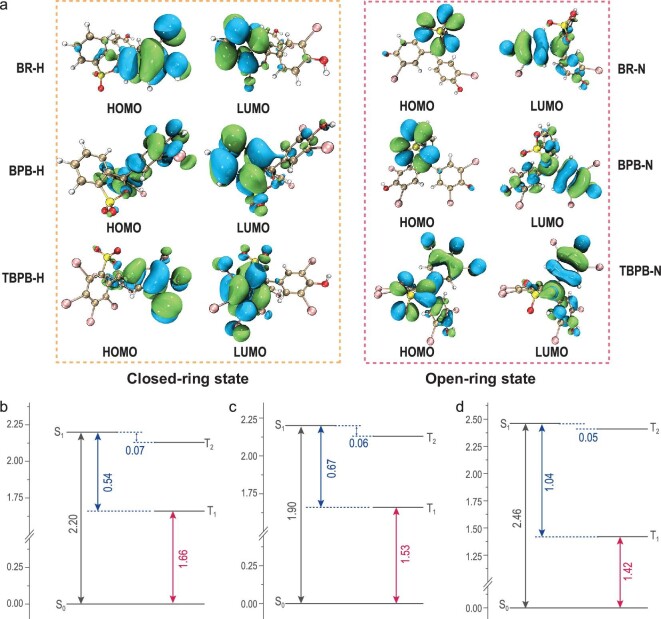
Theoretical analysis. (a) The HOMO orbitals and LUMO orbitals of BR, BPB and TBPB were calculated using DFT by Gaussian 09 in the open-ring states (in the orange box) and closed-ring states (in the red box) (isosurface value = 0.02) (Fig. S11) [41]. The energy diagram (based on the optimized geometry of the T_1_ state) of the excited states S_1_ and T_n_ for (b) BR-N, (c) BPB-N and (d) TBPB-N (n = 1,2; units: eV).

Structural analysis and density functional theory (DFT) were used to analyze the properties of these brominated PSP derivatives in the PVA matrix. DFT calculations calculated the highest occupied molecular orbital (HOMO) and the lowest unoccupied molecular orbital (LUMO) of PSP derivatives in the ground state (S_0_) for both open and closed states. According to the time-dependent DFT (TD-DFT) theoretical analysis, the HOMO and LUMO orbits of PSP derivatives were the main orbits involved in the excitation process of PSP derivatives. Figure 3 presents charge transfer processes during excitation of PSP derivatives, in which the PSP derivatives could be considered as compounds with an electron donor (D)–acceptor (A) structure, whether in the open-ring or closed-ring state. Also, the calculated quantitative data of TD-DFT were in good agreement with the experimental data. Table S2 shows that the energy differences between T_2_ and S_1_ of PSP molecules are small (<0.1 eV), which is conducive to generation of their triplet excited states (Fig. 3b–d). Moreover, introduction of the heavy-atom effect (-Br moieties) of open-ring PSP molecules could effectively promote the spin-orbital coupling (SOC) of the system to promote the ISC process (Table 2), and the spin-orbit matrix elements (<S_1_|H_SO_|T_n_>) [37] would increase significantly with an increasing number of heavy atomatic modifications. According to El-Sayed rules, the n→π^*^ transition of the orange area could further allow the ISC process to induce RTP emission. Existing studies show that organic molecules with small HOMO and LUMO orbital overlaps have a small singlet-triplet energy gap (*▵*E_ST_) because of small exchange energy between them, thus making it easy to induce TADF [38,39]. Based on the DFT analysis, it was also found that minimal overlap between HOMO and LUMO orbitals for PSP molecules was conducive to generating TADF.

**Table 2.** Spin-orbit matrix elements <S_1_|H_SO_|T_n_> (cm^−1^) for the BR-N, BPB-N and TBPB-N (Figs S12–S14) [37].

	<S_1_|H_SO_|T_1_>	<S_1_|H_SO_|T_2_>
BR-N	3.60	1.86
BPB-N	4.11	1.57
TBPB-N	15.36	8.14

The different moieties of PSP derivatives are color-coded in Fig. 1b to analyze generation of near-infrared RTP by doping PSP derivatives in the PVA matrix. It is reasonable for PSP-N molecules to emit red fluorescence in the rigid matrix because of their D-A structures. The ring-opening state of PSP@PVA-N has an apparent D-A structure, in which the orange moiety is the D moiety (Figs 1b, 3 and S10), the dark pink moiety is the A moiety and the sp^2^ hybridized double bond between the D/A moieties could be considered as a π linker. The existence of a π linker can ensure conjugation between the D and A moieties in the PSP-N molecules. In the closed-ring state, the double bond of sp^2^ hybridization was replaced by the orange moiety and became the quaternary carbon of sp^3^ hybridization. Therefore, the π linker between the D and A moieties in PSP-H would be significantly weakened by becoming an σ linker, thus greatly reducing the conjugation effect. The roles of D and A in the closed-ring system were reversed, and the conjunction intensity between D and A was substantially reduced because of the ring-forming of the orange moiety. Thus, the D-A structure provided the open-ring state of PSP@PVA-N films with basic conditions for long-wavelength emission, while the closed-ring state of PSP@PVA-H films without the D-A structure had no long-wavelength emission. Also, the prompted fluorescence wavelength of BPB@PVA-N and BR@PVA-N was similar, yet in contrast, the fluorescence emission of TBPB@PVA-N had a redshift of nearly 40 nm.

Existing studies show that the stronger the electron delocalization ability of the molecule with the D-A structure, the more favorable it is to produce longer-wavelength emission [40]. It can be inferred that the number of the Br atom, as a weak electron-withdrawing group, may effectively shift the emission peak to a longer wavelength as a result of electron delocalization between the D and A moieties of PSP molecules [40]. In addition, the emission peaks of RTP must be located at a longer wavelength than that of fluorescence because of the large Stokes shift of RTP emission. Hence, only a slight redshift was observed between BPB@PVA-N (λ_ex_ = 606 nm, λ_p_ = 793 nm, Φ_RTP_ = 2.6%, τ = 0.58 ms) and BR@PVA-N (λ_ex_ = 603 nm, λ_p_ = 789 nm, Φ_RTP_ = 1.8%, τ = 0.72 ms). The phenomenon of the larger redshift of TBPB@PVA-N (λ_ex_ = 634 nm, λ_p_ = 819 nm, Φ_RTP_ = 3.0%, τ = 0.27 ms) was generated by introduction of a halogen atom, which reduced the strength of the electron delocalization between the electron donor and the acceptor (Fig. 3a).

**Figure 4.**
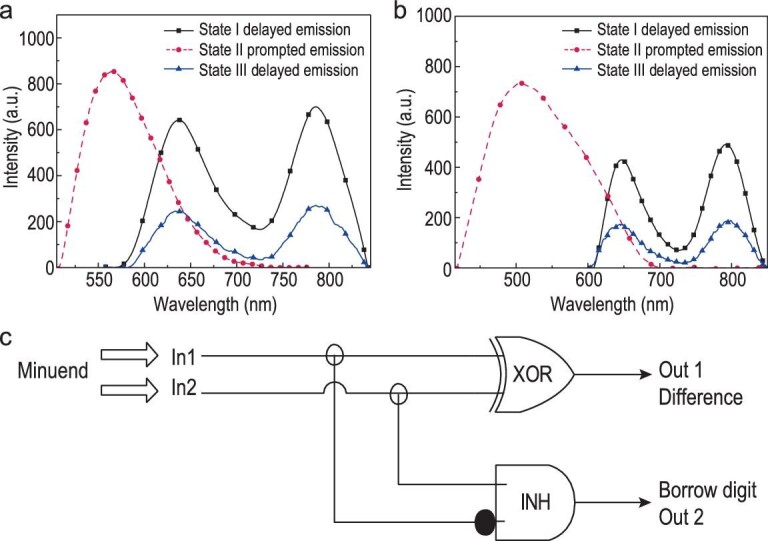
(a) Delayed emission spectra (BR@PVA-N, λ_ex_ = 550 nm) and prompted emission spectra (BR@PVA-H, λ_ex_ = 450 nm) of BR@PVA films after *insitu* acid-base adjustment. (State I and State III: open-ring state; State II: closed-ring state.) (b) Delayed emission spectra (BPB@PVA-N, λ_ex_ = 550 nm) and prompted emission spectra (BPB@PVA-H, λ_ex_ = 450 nm) of BPB@PVA films after *insitu* acid-base adjustment. (c) The logic operation of the half-subtractor was constructed using the reversible properties of the PSP molecules adjusted by acid and base.

Different photophysical properties of PSP derivatives in the PVA matrix were used to construct a half-subtracter, which indicates an advanced logic operation (Fig. 4a–c). The PSP@PVA film could adjust the ON-OFF ring state of PSP molecules *in situ* by acid-base solution dropwise or acid-base vapor fumigation. Furthermore, PSP@PVA-H can be converted to PSP@PVA-N to regenerate RTP in the presence of a proper base (amine gas or ammonia), although RTP will be reduced compared with its original state before vacuum drying to remove water [42,43]. Based on this, an ‘ON-OFF’ RTP switch was constructed. A logic operation with the half-subtractor function and the dual-channel response was developed (Fig. 4c). Compared with the traditional half-subtractor [44,45], no parallel logic gates are needed in this system to respond synchronously to achieve the effect of binary subtraction calculation. In this half-subtractor, both the NIR RTP emission and visible light emission of the system were set as output signals expressed by negative logic, while the acid-base response properties of the system were set as input signals expressed by positive logic. Table 3 is the truth table of the half-subtractor. Of note, PVA with low degree of alcoholysis was prone to crosslinking reaction under alkaline conditions, and the intermolecular hydrogen bonds within PVA would be destroyed to quench emissions of the proposed systems. When the films were adjusted with excessive base, the base would induce crosslinking of the PVA matrix to form a gel, sharply quenching the visible light emission and the NIR RTP emission of the system. For practical application of the aforementioned logic operation, the responsive speed of the system was evaluated. The responsive speed for an individual operation of the system was recorded to be around 2–4 seconds per loop. The components of the responsive speed for this individual operation included the acid/base responsive process and photo-electronic signal responsive process, in which the responsive speed of standalone acid/base response was around 1–2 seconds per loop, and the total responsive speed of the linear programming acid/base response was around 2–4 seconds per loop. Furthermore, the responsive time of the photo-electronic signal responsive process was based on the frequency of electronical signals (ms level). Therefore, the total responsive speed of this logic operation should be around 2–4 seconds.

**Table 3.** Truth table of the demultiplexer half-subtractor.

I1 Acid	I2 Base	O1 NIR	O2 VIS
0	0	0	0
0	1	1	1
1	0	1	0
1	1	0	0

I1 and I2 are the positive logical specified expressions; O1 and O2 are the negative logical specified expressions. NIR represents detection of NIR RTP emission, and VIS represents detection of visible light emission.

## CONCLUSION

In summary, a set of red-light-excited, metal-free RTP films with the highest NIR RTP quantum yield (Φ_RTP_ = 3.0%) and the reddest emission (λ_p_ = 819 nm) were constructed in this research. These films constructed highly efficient near-infrared RTP materials with prevalent raw materials and a convenient preparation process. DFT and TD-DFT calculations were used to analyze the near-infrared RTP emission of PSP derivatives in the PVA matrix, and the structural tunability of PSP derivatives was further used to verify them experimentally. Moreover, a logic operation with half-subtractor function and dual-channel response (visible light emission/NIR RTP emission) was constructed based on these properties. The difficulty in constructing NIR emission lays in the rapid internal conversion between low-energy-level S_1_ and S_0_, which was explored in light of energy-gap law. As the internal conversion process between T_1_ and S_0_ was forbidden (given the changed electron spin state, based on Kasha's rule and the phenomenon in which the energy of T_1_ was lower than that of S_1_), it can be inferred that NIR emission could be designed from a new perspective of a triplet excited state, including enhancing the ISC efficiency in dye molecules with red fluorescence or suppressing the other non-radiative decay process. Last but not least, the short lifetime of the triplet state needs to be re-considered, because its long lifetime can neutralize favorable factors caused by the spin suppression process of internal conversion so as to quench the NIR radiative emission. This advantage may effectively reduce the threshold for constructing metal-free NIR emission systems.

## MATERIALS AND METHODS

Bromophenol red (BR), bromophenol blue (BPB) and tetrabromophenol blue (TBPB) were purchased from Shanghai Macklin Biochemical Co., Ltd. Polyvinyl alcohol (PVA, alcoholysis degree 87%) was purchased from Shanghai Aladdin Bio-Chem Technology Co., Ltd. All solvents were purchased commercially and used without further purification. The UV-Vis absorption spectra were obtained on a Cary 60 (Agilent Technologies) spectrophotometer. Fluorescence, phosphorescence and the lifetime of delayed emission spectra were recorded on an Agilent Cary Eclipse spectrophotometer. Phosphorescence mode; delay time = 0.1 ms; gate time = 2.0 ms. Photoluminescence spectra were recorded on the HORIBA FluoroMax-4 spectrometer. Absolute PL quantum yields were determined with a spectrometer C11347-11 (Hamamatsu, Japan).

### Preparation of BPB@PVA-N (1 wt%) films

Firstly, 1g PVA powder was dissolved in 20 mL water, and 10 mg BPB dye was added into the PVA solution. After the mixture was sonicated for 20 minutes, the corresponding BPB@PVA-N (1 wt%) film was obtained by vacuum distillation. The BPB@PVA-N (1 wt%) film was prepared by tearing the film and drying it in vacuum at 50°C for 24 hours.

### Preparation of BR@PVA-N (1 wt%) and TBPB@PVA-N (1 wt%) films

The preparation process was the same as that for BPB@PVA-N films.

### Preparation of BPB@PVA-H (1 wt%) films

Firstly, 1 g PVA powder was dissolved in 20 mL water, and 10 mg BPB dye was added into the PVA solution. Then, HCl was added to the obtained BPB@PVA aqueous solution to adjust the pH of the solution, and a wide range of pH test paper and pH meter were used to determine the pH of the solution (pH = 1). After the mixture was sonicated for 20 minutes, the corresponding BPB@PVA-H (1 wt%) film was obtained by vacuum distillation. The BPB@PVA-H (1 wt%) film was prepared by tearing the film and drying it in a vacuum at 50°C for 24 hours.

### Preparation of BR@PVA-H (1 wt%) and TBPB@PVA-H (1 wt%) films

The preparation process was the same as that for BPB@PVA-H films.

### Adjusting the pH environment of the PSP@PVA film

HCl was used to reduce the pH environment of the PSP@PVA films. Ammonia water and sodium hydroxide aqueous solution were used to increase the pH of the PSP@PVA films. *Insitu* adjustment of PSP@PVA films was done by spraying an appropriate amount of hydrochloric acid solution or ammonia solution on the films’ surfaces. This process can be effectively monitored through the absorption spectra and the color of the films.

## Supplementary Material

nwab085_Supplemental_File
