# Supplementary material for: Red-light excited efficient metal-free near-infrared room-temperature phosphorescent films
Source: Natl Sci Rev. 2021 May 11;9(2):nwab085. doi: 10.1093/nsr/nwab085 (PMC8866102; doi:10.1093/nsr/nwab085)
Supplement: nwab085_Supplemental_File [file nwab085_supplemental_file.docx]

**Supporting Information**

**Red-light Excited Efficient Near-Infrared Metal-free Room-Temperature Phosphorescent Films**

Siyu Sun, Liangwei Ma, Jie Wang, Xiang Ma* and He Tian

Key Laboratory for Advanced Materials and Feringa Nobel Prize Scientist Joint Research Center, Frontiers Science Center for Materiobiology and Dynamic Chemistry, School of Chemistry and Molecular Engineering, East China University of Science and Technology, Shanghai 200237, China

**Corresponding Author**

*maxiang@ecust.edu.cn

**Contents**

[1. Spectra and Photographes 2](#_Toc68199567)

[2. Theoretical calculations 8](#_Toc68199568)

[3. References 23](#_Toc68199569)

## 1. Spectra and Photographes

***Figure S1.*** XRD spectra of **TBPB@PVA-N**, **BPB@PVA-N**, and **BR@PVA-N.**

***Figure S2.*** XRD spectra of **TBPB@PVA-H**, **BPB@PVA-H**, and **BR@PVA-H.**

***Figure S3.*** UV absorption spectra of **BR@PVA-N** and **BR@PVA-H**

***Figure S4.*** UV absorption spectra of **BPB@PVA-N** and **BPB@PVA-H**

***Figure S5.*** UV absorption spectra of **TBPB@PVA-N** and **TBPB@PVA-H**


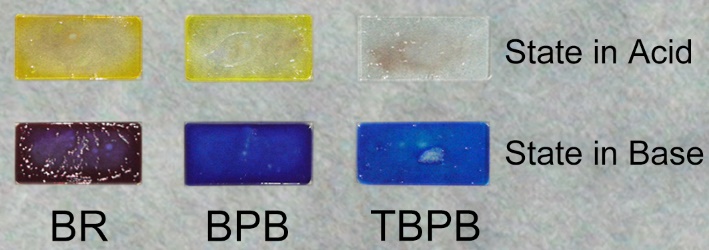


***Figure S6.*** The color changes of **BR@PVA**, **BPB@PVA**, and **TBPB@PVA** under different states (acid state and base state).

***Figure S7.*** The TADF (black dot) and RTP (red dot) lifetime curve of **BR@PVA-N** at an excitation wavelength of 600 nm. (Insert: Enlarged view of TADF and RTP lifetime scatter plot from 0 ms to 5 ms)

***Figure S8.*** The TADF (black dot) and RTP (red dot) lifetime curve of **BPB@PVA-N** at an excitation wavelength of 600 nm. (Insert: Enlarged view of TADF and RTP lifetime scatter plot from 0 ms to 5 ms)

***Figure S9.*** The TADF (black dot) and RTP (red dot) lifetime curve of **TBPB@PVA-N** at an excitation wavelength of 600 nm. (Insert: Enlarged view of TADF and RTP lifetime scatter plot from 0 ms to 5 ms)

***Table S1.*** Lifetimes Fitting Variance of TADF and RTP of PSP@PVA-N Systems (r^2^ is Fitting Variance)

|  | BR_TADF | BR_RTP | BPB_TADF | BPB_RTP | TBPB_TADF | TBPB_RTP |
| --- | --- | --- | --- | --- | --- | --- |
| τ (μs) | 292 | 722 | 354 | 580 | 225 | 278 |
| r^2^ | 0.99998 | 0.99998 | 0.99999 | 0.99999 | 0.99999 | 1 |

***Figure S10.*** Phosphorescence and Fluorescence emission spectra of **TBPB@PVA-H** (λ_ex(f)_ = 425 nm, λ_ex(p)_ = 600 nm).

## 2. Theoretical calculations

General Density functional theory (DFT) (46) calculations were performed with the Gaussian 09 (Revision E.01) software package. The ground-state (S_0_) was optimized with the B3LYP5 and 6-31G(d,p) basis. These theoretical simulation calculations were simplified and approximated just to figure out the general law of the research system.


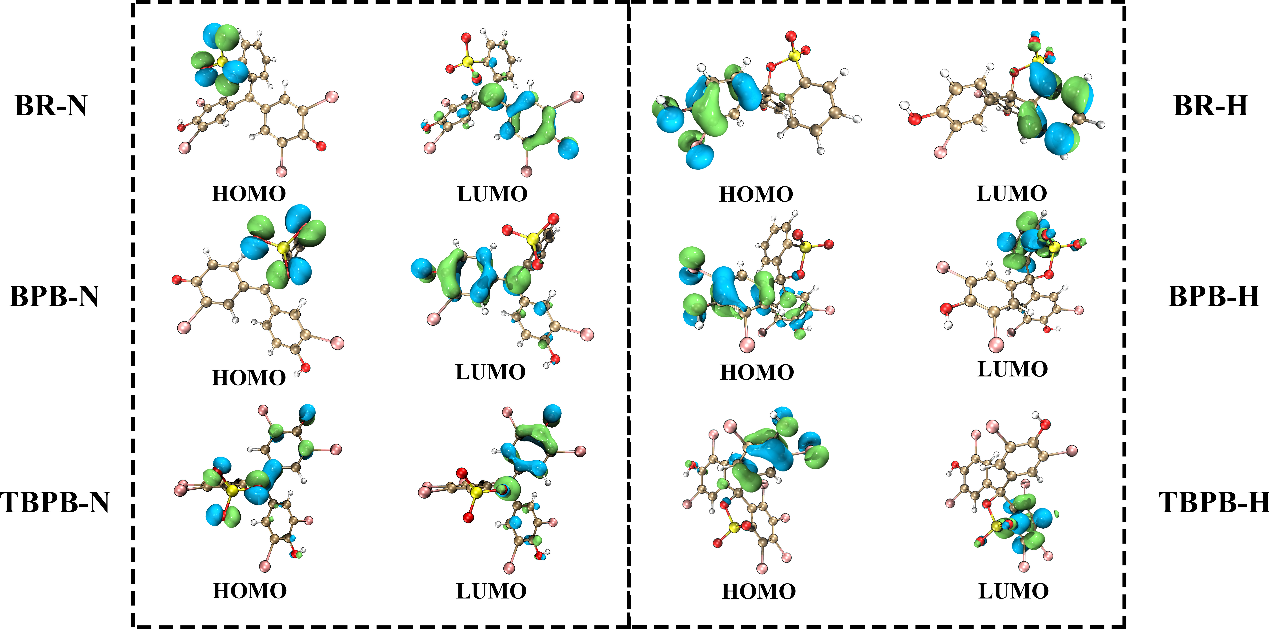


***Figure S11.*** HOMO and LUMO orbitals of **PSP** derivatives under acidic and neutral conditions (isosurface value = 0.05).

**Table. S2** the detailed information of energy levels of the excited state was calculated by TD-DFT in Gaussian 09. The energy difference between T_1_ and S_1_ was large (> 0.3 eV), and the energy difference between T_2_ and S_1_ was small (< 0.1 eV).

|  | **T_1_ (eV)** | **T_2_ (eV)** | **S_1_ (eV)** | ***△*E_ST_ (eV)** |
| --- | --- | --- | --- | --- |
| **BR** | 1.66 | 2.13 | 2.20 | 0.07 |
| **BPB** | 1.53 | 1.84 | 1.90 | 0.06 |
| **TBPB** | 1.42 | 2.41 | 2.46 | 0.05 |

**BR-N**

Excitation energies and oscillator strengths:

Excited State 1: Triplet-A 1.6599 eV 746.95 nm f=0.0000 <S**2>=2.000

118 ->127 -0.12326

120 ->127 0.12265

124 ->127 -0.23183

125 ->127 0.61001

126 ->127 0.14796

125 <-127 0.12660

This state for optimization and/or second-order correction.

Total Energy, E(TD-HF/TD-KS) = -6648.31436249

Copying the excited state density for this state as the 1-particle RhoCI density.

Excited State 2: Triplet-A 2.1231 eV 583.97 nm f=0.0000 <S**2>=2.000

125 ->127 -0.13741

126 ->127 0.68883

Excited State 3: Singlet-A 2.1957 eV 564.66 nm f=0.0121 <S**2>=0.000

126 ->127 0.70289

Excited State 4: Triplet-A 2.5943 eV 477.90 nm f=0.0000 <S**2>=2.000

121 ->127 0.65822

121 ->136 0.10351

123 ->127 0.18056

Excited State 5: Triplet-A 2.7827 eV 445.55 nm f=0.0000 <S**2>=2.000

119 ->127 0.26670

124 ->127 0.57485

125 ->127 0.26340

Excited State 6: Singlet-A 2.8403 eV 436.51 nm f=0.0210 <S**2>=0.000

124 ->127 0.55761

125 ->127 0.42241

Excited State 7: Singlet-A 2.9788 eV 416.22 nm f=0.0027 <S**2>=0.000

121 ->127 0.64978

123 ->127 0.22194

124 ->127 0.10970

Excited State 8: Triplet-A 2.9821 eV 415.76 nm f=0.0000 <S**2>=2.000

119 ->127 0.55015

120 ->127 0.11450

123 ->127 0.19627

124 ->127 -0.27517

125 ->133 0.10463

Excited State 9: Singlet-A 3.1584 eV 392.55 nm f=0.0997 <S**2>=0.000

121 ->127 -0.11180

122 ->127 0.14168

123 ->127 0.49910

124 ->127 -0.28537

125 ->127 0.34872

Excited State 10: Triplet-A 3.1650 eV 391.74 nm f=0.0000 <S**2>=2.000

119 ->127 -0.17118

121 ->127 -0.15900

122 ->127 0.16290

123 ->127 0.62489

125 ->127 0.11407

Excited State 11: Triplet-A 3.2742 eV 378.67 nm f=0.0000 <S**2>=2.000

117 ->127 -0.13525

118 ->127 -0.24498

120 ->127 0.38431

120 ->128 -0.12917

122 ->127 -0.31298

124 ->127 0.16429

125 ->127 -0.11027

125 ->129 0.13913

Excited State 12: Singlet-A 3.3871 eV 366.05 nm f=0.2797 <S**2>=0.000

118 ->127 -0.18338

119 ->127 -0.12129

121 ->127 -0.19227

122 ->127 -0.12936

123 ->127 0.43857

124 ->127 0.27029

125 ->127 -0.34120

Excited State 13: Triplet-A 3.4140 eV 363.16 nm f=0.0000 <S**2>=2.000

120 ->127 0.24171

122 ->127 0.59336

123 ->127 -0.12543

125 ->129 0.10272

Excited State 14: Singlet-A 3.4143 eV 363.13 nm f=0.0360 <S**2>=0.000

122 ->127 0.67494

125 ->127 -0.15017

Excited State 15: Triplet-A 3.5165 eV 352.58 nm f=0.0000 <S**2>=2.000

118 ->127 0.38882

118 ->130 -0.23905

124 ->129 0.13641

124 ->131 -0.14444

124 ->132 0.12777

125 ->128 0.15620

125 ->129 0.15551

Excited State 16: Singlet-A 3.7268 eV 332.68 nm f=0.0197 <S**2>=0.000

118 ->127 -0.13171

119 ->127 0.64052

120 ->127 0.24574

Excited State 17: Triplet-A 3.7296 eV 332.43 nm f=0.0000 <S**2>=2.000

115 ->127 -0.10604

117 ->127 0.16127

117 ->128 0.10316

118 ->127 0.31091

120 ->127 0.45572

120 ->128 0.11930

125 ->129 -0.16019

Excited State 18: Singlet-A 3.7956 eV 326.65 nm f=0.0078 <S**2>=0.000

118 ->127 0.25278

120 ->127 0.28054

126 ->128 0.57701

126 ->129 0.10943

Excited State 19: Singlet-A 3.8062 eV 325.74 nm f=0.0243 <S**2>=0.000

118 ->127 0.42004

120 ->127 0.39253

126 ->128 -0.37519

Excited State 20: Singlet-A 4.0053 eV 309.55 nm f=0.1556 <S**2>=0.000

114 ->127 -0.13485

115 ->127 0.14536

117 ->127 -0.11207

118 ->127 0.38709

119 ->127 0.20681

120 ->127 -0.37909

125 ->127 -0.17617

126 ->129 0.19320

**BPB-N:**

Excitation energies and oscillator strengths:

Excited State 1: Triplet-A 1.5334 eV 808.54 nm f=0.0000 <S**2>=2.000

153 ->161 0.10220

159 ->161 0.63125

160 ->161 -0.27807

159 <-161 0.12786

This state for optimization and/or second-order correction.

Total Energy, E(TD-HF/TD-KS) = -11790.5297636

Copying the excited state density for this state as the 1-particle RhoCI density.

Excited State 2: Triplet-A 1.8443 eV 672.25 nm f=0.0000 <S**2>=2.000

159 ->161 0.27252

160 ->161 0.64782

Excited State 3: Singlet-A 1.9019 eV 651.89 nm f=0.0110 <S**2>=0.000

160 ->161 0.70072

Excited State 4: Triplet-A 2.5504 eV 486.13 nm f=0.0000 <S**2>=2.000

157 ->161 -0.18213

158 ->161 0.66759

Excited State 5: Singlet-A 2.6236 eV 472.56 nm f=0.0014 <S**2>=0.000

157 ->161 -0.13108

158 ->161 0.68771

Excited State 6: Triplet-A 2.6575 eV 466.54 nm f=0.0000 <S**2>=2.000

153 ->161 0.25533

154 ->161 0.29788

157 ->161 0.53354

158 ->161 0.17540

Excited State 7: Triplet-A 2.7183 eV 456.11 nm f=0.0000 <S**2>=2.000

152 ->161 -0.16030

153 ->161 0.19252

154 ->161 0.50769

157 ->161 -0.37718

Excited State 8: Triplet-A 2.7978 eV 443.15 nm f=0.0000 <S**2>=2.000

141 ->161 0.10470

152 ->161 -0.18013

153 ->161 -0.19614

155 ->161 0.60153

159 ->165 0.10210

Excited State 9: Singlet-A 2.8740 eV 431.40 nm f=0.0213 <S**2>=0.000

156 ->161 0.10259

157 ->161 0.67459

158 ->161 0.13423

Excited State 10: Singlet-A 3.0019 eV 413.01 nm f=0.2835 <S**2>=0.000

153 ->161 -0.28417

154 ->161 -0.38824

156 ->161 0.10676

159 ->161 0.48554

Excited State 11: Triplet-A 3.0164 eV 411.03 nm f=0.0000 <S**2>=2.000

153 ->161 -0.15890

155 ->161 -0.13525

156 ->161 0.63875

Excited State 12: Singlet-A 3.0970 eV 400.34 nm f=0.2071 <S**2>=0.000

154 ->161 -0.30271

156 ->161 0.48391

159 ->161 -0.38331

Excited State 13: Singlet-A 3.0990 eV 400.08 nm f=0.1211 <S**2>=0.000

152 ->161 -0.10795

153 ->161 0.10711

154 ->161 0.36918

156 ->161 0.48987

157 ->161 -0.13273

159 ->161 0.26695

Excited State 14: Triplet-A 3.1223 eV 397.09 nm f=0.0000 <S**2>=2.000

152 ->161 0.11309

153 ->161 0.48147

154 ->161 -0.19855

155 ->161 0.19883

156 ->161 0.27563

157 ->161 -0.11444

158 ->161 -0.10118

159 ->161 -0.10491

159 ->164 -0.10442

Excited State 15: Triplet-A 3.3277 eV 372.58 nm f=0.0000 <S**2>=2.000

151 ->161 -0.16811

152 ->161 0.57495

153 ->161 -0.10869

154 ->161 0.19998

155 ->161 0.13618

Excited State 16: Singlet-A 3.3673 eV 368.20 nm f=0.0032 <S**2>=0.000

153 ->161 -0.16195

155 ->161 0.67436

Excited State 17: Triplet-A 3.4548 eV 358.88 nm f=0.0000 <S**2>=2.000

148 ->161 -0.20513

150 ->161 -0.12455

151 ->161 0.46820

152 ->161 0.19949

153 ->161 -0.13004

154 ->161 0.16067

158 ->165 -0.10409

Excited State 18: Singlet-A 3.5516 eV 349.09 nm f=0.0212 <S**2>=0.000

152 ->161 0.67499

154 ->161 0.14569

Excited State 19: Singlet-A 3.5591 eV 348.36 nm f=0.0797 <S**2>=0.000

151 ->161 -0.18456

153 ->161 0.57258

154 ->161 -0.27184

155 ->161 0.15371

159 ->161 0.12456

Excited State 20: Singlet-A 3.7059 eV 334.56 nm f=0.0114 <S**2>=0.000

160 ->162 0.22208

160 ->163 0.66124

**TBPB-N**

Excited State 1: Triplet-A 1.4215 eV 872.21 nm f=0.0000 <S**2>=2.000

227 ->229 -0.35294

228 ->229 0.60215

228 <-229 0.13608

This state for optimization and/or second-order correction.

Total Energy, E(TD-HF/TD-KS) = -22074.9252142

Copying the excited state density for this state as the 1-particle RhoCI density.

Excited State 2: Triplet-A 2.4110 eV 514.24 nm f=0.0000 <S**2>=2.000

227 ->229 0.60112

228 ->229 0.35999

Excited State 3: Singlet-A 2.4595 eV 504.10 nm f=0.0056 <S**2>=0.000

227 ->229 0.55195

228 ->229 0.43718

Excited State 4: Triplet-A 2.6583 eV 466.40 nm f=0.0000 <S**2>=2.000

221 ->229 0.18793

222 ->229 0.43616

223 ->229 0.40054

224 ->229 -0.16871

225 ->229 -0.19202

226 ->229 -0.11171

Excited State 5: Triplet-A 2.7431 eV 451.98 nm f=0.0000 <S**2>=2.000

222 ->229 0.23383

224 ->229 0.62174

228 ->236 0.11501

Excited State 6: Singlet-A 3.0048 eV 412.62 nm f=0.1306 <S**2>=0.000

221 ->229 0.14397

222 ->229 0.38422

223 ->229 0.38480

225 ->229 -0.13800

226 ->229 -0.14556

227 ->229 0.22205

228 ->229 -0.27038

Excited State 7: Triplet-A 3.0094 eV 411.98 nm f=0.0000 <S**2>=2.000

219 ->229 -0.10513

220 ->229 0.13105

221 ->229 0.26817

225 ->229 0.41708

226 ->229 0.36035

Excited State 8: Singlet-A 3.0692 eV 403.96 nm f=0.3815 <S**2>=0.000

221 ->229 0.14902

222 ->229 0.23137

223 ->229 0.17115

225 ->229 -0.19814

227 ->229 -0.36730

228 ->229 0.45076

Excited State 9: Triplet-A 3.1961 eV 387.92 nm f=0.0000 <S**2>=2.000

223 ->231 -0.10459

225 ->229 -0.39344

225 ->232 -0.12076

226 ->229 0.47826

226 ->232 0.19673

Excited State 10: Singlet-A 3.2533 eV 381.11 nm f=0.0163 <S**2>=0.000

225 ->229 -0.17112

226 ->229 0.66928

228 ->229 -0.10200

Excited State 11: Triplet-A 3.3227 eV 373.14 nm f=0.0000 <S**2>=2.000

218 ->231 -0.12125

222 ->231 0.15779

223 ->231 -0.21979

225 ->229 0.16807

225 ->231 0.10937

225 ->232 -0.22121

226 ->229 -0.26061

226 ->232 0.35640

Excited State 12: Singlet-A 3.3355 eV 371.71 nm f=0.0009 <S**2>=0.000

222 ->229 0.15426

224 ->229 0.66288

225 ->229 0.16076

Excited State 13: Singlet-A 3.3454 eV 370.61 nm f=0.0826 <S**2>=0.000

222 ->229 0.19224

224 ->229 -0.20989

225 ->229 0.60977

226 ->229 0.14047

Excited State 14: Triplet-A 3.3823 eV 366.57 nm f=0.0000 <S**2>=2.000

220 ->229 -0.11206

221 ->229 0.40441

222 ->229 -0.39996

223 ->229 0.20679

224 ->229 0.16902

225 ->229 -0.17956

Excited State 15: Triplet-A 3.3993 eV 364.73 nm f=0.0000 <S**2>=2.000

217 ->229 0.17569

218 ->229 0.11652

221 ->229 -0.25377

222 ->229 -0.21319

223 ->229 0.45940

225 ->229 0.12688

226 ->229 0.19261

Excited State 16: Singlet-A 3.4570 eV 358.64 nm f=0.0108 <S**2>=0.000

222 ->229 -0.43793

223 ->229 0.53592

Excited State 17: Triplet-A 3.5329 eV 350.94 nm f=0.0000 <S**2>=2.000

219 ->229 -0.15777

220 ->229 0.44318

220 ->234 0.11317

221 ->229 -0.22931

223 ->229 0.17098

225 ->229 -0.15093

228 ->230 -0.28703

Excited State 18: Singlet-A 3.5812 eV 346.21 nm f=0.0014 <S**2>=0.000

221 ->229 0.19052

227 ->230 0.28234

228 ->230 0.60978

Excited State 19: Singlet-A 3.6033 eV 344.09 nm f=0.0204 <S**2>=0.000

219 ->229 0.16821

221 ->229 0.58738

222 ->229 -0.14902

227 ->230 -0.24319

228 ->230 -0.10553

Excited State 20: Singlet-A 3.6463 eV 340.03 nm f=0.0045 <S**2>=0.000

221 ->229 0.14044

227 ->230 0.59223

228 ->230 -0.32949

TD-DFT calculations for **BR-H**

Excited State 1: Singlet-A 4.4560 eV 278.24 nm f=0.0090 <S**2>=0.000

126 ->127 0.69891

Excited State 2: Singlet-A 4.5716 eV 271.20 nm f=0.0032 <S**2>=0.000

125 ->127 0.68503

125 ->128 -0.13768

Excited State 3: Singlet-A 4.6011 eV 269.47 nm f=0.0614 <S**2>=0.000

126 ->128 0.67659

TD-DFT calculations for **BPB-H**

Excited State 1: Singlet-A 3.9561 eV 313.40 nm f=0.0024 <S**2>=0.000

227 ->229 0.11952

228 ->229 0.69066

Excited State 2: Singlet-A 3.9866 eV 311.01 nm f=0.0067 <S**2>=0.000

227 ->229 0.69039

228 ->229 -0.12032

Excited State 3: Singlet-A 4.1112 eV 301.58 nm f=0.0090 <S**2>=0.000

227 ->230 0.27255

228 ->230 0.64044

TD-DFT calculations for **TBPB-H**

Excited State 1: Singlet-A 3.9561 eV 313.40 nm f=0.0024 <S**2>=0.000

227 ->229 0.11952

228 ->229 0.69066

Excited State 2: Singlet-A 3.9866 eV 311.01 nm f=0.0067 <S**2>=0.000

227 ->229 0.69039

228 ->229 -0.12032

Excited State 3: Singlet-A 4.1112 eV 301.58 nm f=0.0090 <S**2>=0.000

227 ->230 0.27255

228 ->230 0.64044


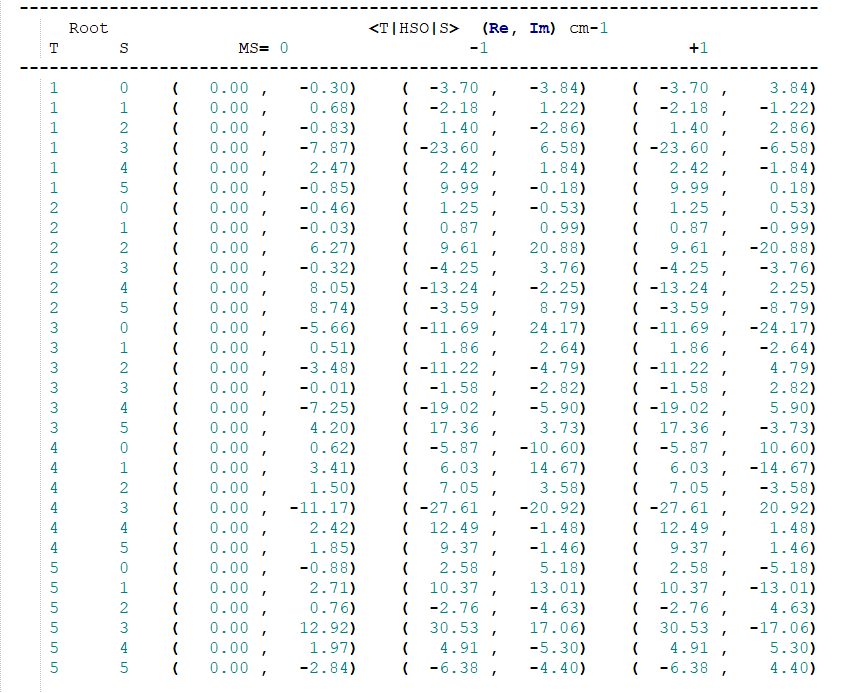


**Figure S12.** **BR**'s spin-orbit coupling matrix caculated with program ORCA6(Version 4.1.0).


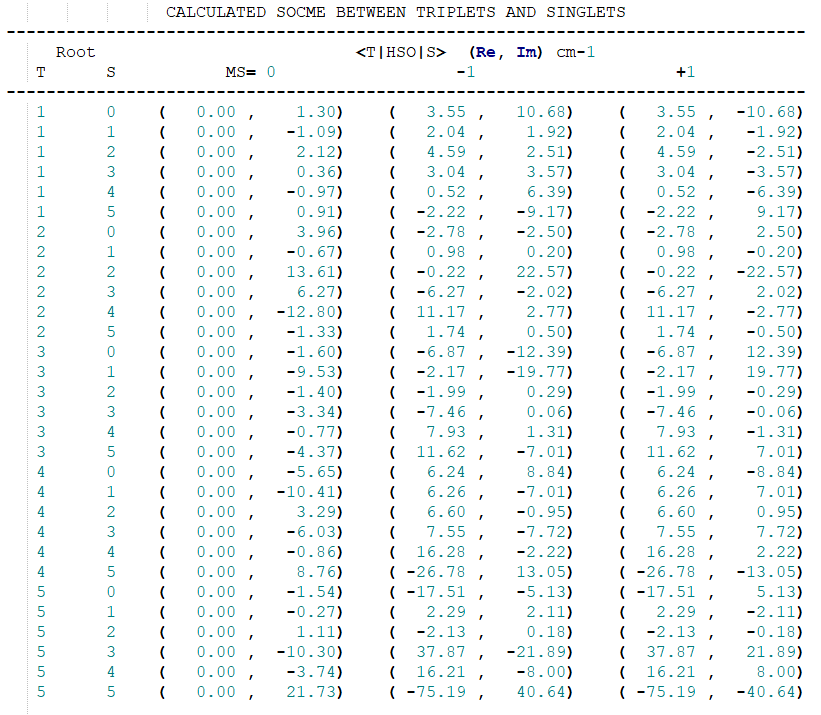


**Figure S13.** **BPB**'s spin-orbit coupling matrix caculated with program ORCA6(Version 4.1.0).


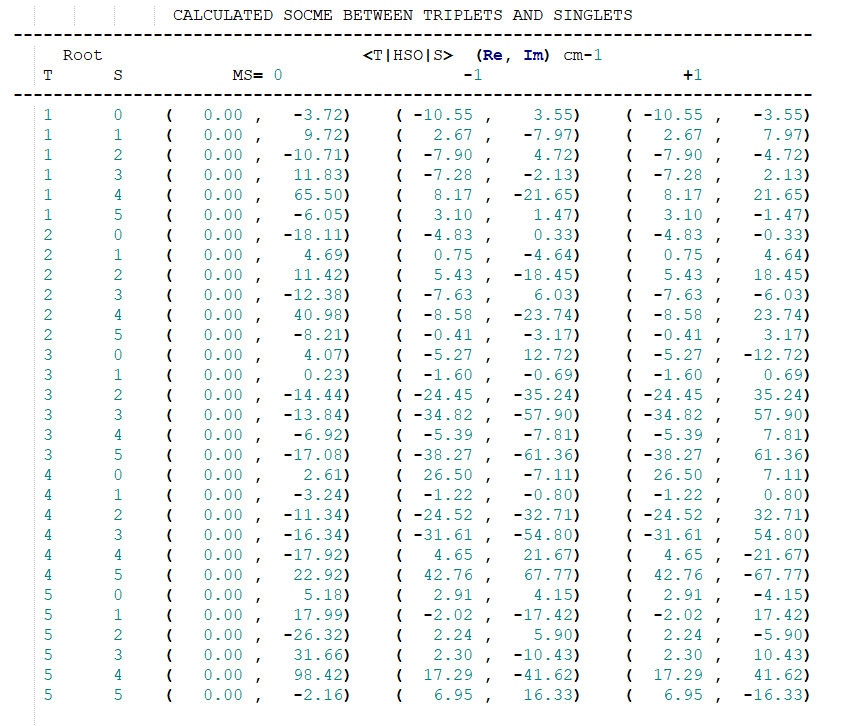


**Figure S14.** **TBPB**'s spin-orbit coupling matrix caculated with program ORCA6(Version 4.1.0) .

## 3. References

1. Runge, E.; Gross, E. K. U., Density-Functional Theory for Time-Dependent Systems. *Phys. Rev. Lett.* **1984,** *52* (12), 997-1000.
